# Young people’s perspectives on the adoption of preventive measures for HIV/AIDS, malaria and family planning in South-West Uganda: focus group study

**DOI:** 10.1186/1471-2458-12-1022

**Published:** 2012-11-22

**Authors:** Jonathan Graffy, Clare Goodhart, Karen Sennett, Gloria Kamusiime, Herbert Tukamushaba

**Affiliations:** 1 Department of Public Health & Primary Care, University of Cambridge, Cambridge, UK; 2 Lensfield Medical Practice, Cambridge, UK; 3 Killick Street Health Centre, London, UK; 4 Volunteer Uganda, Kanungu, Uganda; 5 Great Lakes Regional College, Kanungu, Uganda

## Abstract

**Background:**

Despite the possibility of preventing many cases of HIV, malaria and unplanned pregnancy, protective measures are often not taken by those at risk in Uganda. The study aim was to explore young people’s perspectives on the reasons why this is so.

**Methods:**

Focus groups were conducted with 100 secondary school and college students in Kanungu, Uganda in 2011. Three parallel groups considered HIV, malaria and family planning, and common messages were then explored jointly in a workshop based on the RE-AIM framework (Reach, Effectiveness, Adoption, Implementation and Maintenance).

**Results:**

Participants identified various reasons why preventive action was not always taken. They worried about the effectiveness and side effects of several key interventions: condoms, antiretroviral treatment, various contraceptives and impregnated mosquito nets. Cost, rural isolation and the quality and availability of health services also limited the extent to which people were able to follow health advice. Although there was respect for policy supporting abstinence and fidelity, it was seen as hard to follow and offering inadequate protection when gender imbalance put pressure on women to have sex.

**Conclusions:**

There is an opportunity to improve the uptake of preventive measures by tackling the misconceptions and fears that participants reported with clear, evidence-based messages. This should be done in a way that encourages more open communication about reproductive health between men and women, that reaches out to isolated communities, that draws on both voluntary and government services and enlists young people so that they can shape their future.

## Background

The people of Uganda face multiple health problems and the average life expectancy for the country’s 33 million people is 53 years [1]. Amongst adults aged 15 to 49, 6.5% carry the HIV virus and although this has fallen from the estimated prevalence of 15% in 1995, across the population as a whole 1.2 million still have HIV [2,3]. Many children die of malaria and malnutrition is also a major concern [1]. On average, each Ugandan woman gives birth to 6.3 children and, as a result, the population is growing at a rate of 3.2% a year with 49% being under the age of 18 [4]. As many Ugandans rely on subsistence farming, they worry about the implications for their children of the fragmentation of their small plots of land.

There are a range of policies and programmes to address these challenges to public health. These include government health services, accounting for about 20% of total expenditure on health [3], and non-government organisations, some of which are aligned to religious groups. Government health services in Uganda are officially free, although the quality of these is often criticised and free services are not always available [5-7]. For example, in 2009 only half of those with advanced HIV infection who should have received antiretroviral therapy according to the 2006 WHO guidelines did so [3]. There are particular difficulties in delivering health programmes in rural areas because of the widely dispersed population and undeveloped transport infrastructure.

This report is based on a visit to carry out a health needs assessment on behalf of CHIFCOD, (Child to Family Community Development) a local not-for-profit organisation established in 1999 in Kanungu district, South-West Uganda. Situated at an altitude of around 1,600 m, the undulating green hills are mainly farmland, with forest covering just over a quarter of the area. From conversations with community leaders, visits to educational establishments (2 primary schools, 2 secondary schools and the regional college) and health facilities (drugstores, 3 local health centres and a teaching hospital) it became apparent that although there are detailed national policies to address HIV/AIDS, malaria and population growth, many of these policies are poorly implemented.

The Ugandan Demographic and Health Survey (DHS), conducted in 2006, found that although 90% of adults were aware of the components of the national ABC strategy to Abstain from sex before marriage and Be faithful to one’s partner, only 70% were aware of the value of Condoms [8]. Worryingly, only 56% of sexually active unmarried men reported that they had used a condom when they last had intercourse; for unmarried women the figure was even lower at 39%. When asked whether they had used a condom when they first had sex, 28% of those aged 15 – 24 years who had ever had sex said they had done so on the first occasion.

Similarly, although 97% of Ugandan adults said they knew about family planning, only 52% of married women had ever used a method. Furthermore, despite the fact that 24% of married women were currently using some form of family planning, it was notable that a further 41% of married women could be considered to have an unmet need, in that they wanted to delay a further child for two or more years, or regarded their family as complete, but were not using contraception [8].

Cultural factors are important in shaping sexual behaviour. Many Ugandans are religious, and faith-based organisations played an important role in the community mobilisation that reversed the first wave of the HIV epidemic [2,9]. At that time, public health campaigns emphasised fidelity, and although the number of reported sexual partners fell and the age of first intercourse rose, concerns remain about how unequal relations between men and women affect reproductive health. The practice of paying a “Bride Price”, refundable if the marriage fails, has been seen by some as treating women as commodities and one of the factors contributing the widespread prevalence of forced sex in marriage [10-12]. Polygamy is not uncommon and for vulnerable women, sex as a transaction for presents, money or security is often a part of life [13].

Although malaria is common, with recent data showing that the condition accounts for around 42,000 deaths annually (126 per 100,000) [14], only 10% of respondents in the 2006 DHS said they had slept under an insecticide-treated net on the previous evening [8]. Indeed, only 34% of households owned any type of mosquito net, although the preliminary findings of the 2011 survey show that this had increased to 74% [15]. Given that insecticide-treated nets have been shown to reduce episodes of malaria by half and deaths from malaria in children by one fifth, increasing their use could make a major difference to public health [16,17].

Across these diverse public health concerns, there is a recurring theme of good intentions not leading to the actions needed to protect health. We therefore sought to explore the gap between public policy and patterns of behaviour in Ugandan society. To investigate this, we organised a workshop with focus groups for young people to discuss their intentions and the barriers they perceived to implementing them with regard to HIV/AIDS, malaria and family planning.

## Methods

Participants were recruited through student societies at the regional college and local secondary schools. This approach was taken in order to select young people who might subsequently act as change agents. The workshop was held as part of a one-day conference in November 2011 on the role of young people in tackling HIV/AIDS and malaria, organised jointly by Great Lakes Regional College (a voluntary-aided vocational college, accredited by the National Council for Higher Education), CHIFCOD and a church-linked organisation which provides counselling and advice on HIV/AIDS (Mend the Broken Hearts, Uganda).

Overall 100 young people took part, (46 female and 54 male). Of these 69 were students at the regional college and 31 were from 6 different secondary schools. 8 staff and 4 representatives of local organisations also attended and participated in the discussions. The mean age of college students was 22 and of school students was 17.

### Workshop and focus groups

The workshop was entitled “Good intentions, but not enough action”. Initially the researchers presented information from the 2006 DHS [8] and more recent health data from UNICEF [1]. Drawing on these reports about the uptake of public health interventions for malaria, HIV/AIDS and family planning, the researchers posed the question as to why the uptake of these interventions was not higher. Participants then split into three topic-based focus groups to discuss the reasons for this.

Focus group methods were adopted in order to prompt discussion and encourage the young people to generate solutions to problems identified [18]. During these groups, the RE-AIM framework, developed by Glasgow et al., was used to structure the discussion [19]. This is a widely-adopted approach to evaluate the effectiveness, transferability and impact of public health interventions when applied in uncontrolled everyday settings. It was selected because it allowed factors operating at both the individual and organisational level to be considered. At the beginning of the session, the facilitators explained the meaning of the five RE-AIM domains (Reach, Efficacy, Adoption, Implementation and Maintenance) and each group collected the main points of their discussion on five flip-charts, one for each domain.

The groups discussing HIV and family planning spent part of the time discussing the topic café-style, in mixed sex groups of six, one of whom took notes, whereas the malaria group was conducted as a single group discussion. During the focus groups, the facilitators first asked participants to share their understanding of the topics and preventive measures that people might adopt. Then the groups discussed the reasons these approaches were not implemented more effectively. The groups then used the RE-AIM framework and flip-charts to structure their discussion and prepare a summary which was fed back to the whole group at a plenary [20].

Detailed notes were taken during the workshop and all authors met over the next day to analyse these thematically, both by topic and across the five RE-AIM domains. The analysis was undertaken using a Framework approach, a qualitative method developed for applied social policy research where the main research questions are known in advance [21]. In conducting the analysis, the researchers considered both the content recorded in the notes and flip charts, and also the group interactions observed [18]. In order to check the validity of the findings, these were discussed further with community leaders and subsequently, some additional points were added [22]. These are reported separately, after the report on the perspectives of the young people.

This work, including the arrangements to obtain consent and protect confidentiality, was approved by the Founding Director of Great Lakes Regional College, Kanungu, Uganda who considered that external ethical review was not necessary. The purpose of the workshop was explained verbally and participation was voluntary. Consent was obtained verbally and none of the participants were under the age of 16. No personal identifiers were retained by the researchers and individual participants were not identifiable in the records kept from the focus group discussions. The research was conducted in accordance with the tenets of the Declaration of Helsinki.

## Results

### HIV focus group

The majority of the participants had themselves had an HIV test and also knew someone who had HIV or AIDS. They asked detailed questions and were eager to understand ways the virus could be transmitted, what could help prevent HIV and the benefits of early diagnosis and treatment. During this discussion, they appeared to change their attitude to a diagnosis of HIV, moving from viewing it as a death sentence to an opportunity for early treatment. The group then discussed ways to close the gap between policy intentions and patterns of behaviour, with suggestions for topics that should be addressed (see Table 1). They were keen to take on the role of change agents, but were very aware of the difficulty of following the ABC policy, both for themselves personally, as well as for other people.

**Table 1:** Barriers to prevention and implementation strategies that participants suggested to address these

	**Reach**	**Efficacy**	**Adoption**	**Implementation**	**Maintenance**
**Malaria**	Village shops have no nets.	Doubts about effectiveness of nets	Cost of nets	Groups (for discussion & practical action)	Net replacement & retreatment with insecticide
	Mass media does not reach rural areas.		Feeling too hot in bed	Advice in clinics	
	Mosquito control harder in marshy areas		Reactions to chemicals	Churches	
			Various control measures used but gaps in knowledge about reservoirs for breeding	Mass media	
**HIV/AIDS**	Condom availability	Lack of confidence in condoms	Cost of condoms	Test and treat early	Reliable condom supply
	Limited knowledge	Limited experience of people improving with treatment	Moral views on abstinence	Publicise facts on transmission	
		Lack of confidence in health services	Peer & family pressure	Empower people to discuss HIV risk, say no and defer intercourse until tested	
			Self-control a challenge	How can we help people to be faithful?	
			Alcohol		
			Fear of positive results may discourage testing		
**Family Planning**	Transport limited	Doubts about effectiveness	Cost	Facilitate opening the topic for discussion	If side effects, people tend to stop, rather than try alternatives.
		Not all methods available	Worry about side effects (infection, bleeding, sterility)	Train facilitators	Reliable programme of clinics
		Limited knowledge	Men & women may have different ideas on ideal family size	Use visual aids & drama	
			Religious constraints		

### Malaria

All but one of the participants in the malaria group had themselves had malaria. Early in the discussion it became clear that they saw impregnated nets as merely one of a number of approaches to malaria prevention. Most families cut down bushes around their houses and closed their doors and windows in the evening, but only four of the 30 slept under an impregnated net. Five changed into longer sleeves and trousers in the evening, three had actively dealt with stagnant water and three had covered compost. One used repellent coils. They proposed a range of community-based approaches to raise awareness of these risks and how to tackle them.

### Family planning

Working in small groups, the young people were first asked how many siblings they had and how many children they themselves hoped to have. This revealed that they came from families with a mean of 6.6 children, whilst the mean of their ideal family sizes was 2.9. The Ugandan co-facilitator then led a discussion about the difficulties faced by big families. Suggested challenges included inadequate food, lack of basic home facilities, domestic violence, inability to afford good quality education and future land fragmentation.

The groups then considered reasons for the gap between peoples’ intentions and the reality of the way family planning was used. Of potential contraceptive methods, they were aware that condoms, Depo-Provera (Depot medroxyprogesterone acetate), combined oral contraception and implants were available, although they understood that women would need to travel to another district for implants. There was a sense that better contraceptive methods were available in richer countries while those available locally were thought to have major side effects, reported below.

### Findings grouped by theme

#### Reach

Participants identified particular problems for the many isolated villages where people were less aware of how to address health problems: few families had access to mass media and few leaflets were available; schools were less well developed with consequently lower child and adult literacy levels and it was sometimes hard to explain technical concepts in the local language. Impregnated nets were unavailable in rural village shops, health services outreach was limited to basic immunisations and family planning services rarely extended to isolated villages. Suggestions to address this included training local facilitators and providing transport for outreach services.

#### Efficacy

Some participants expressed a lack of confidence in the various interventions available. For example, they questioned whether it was worth using a mosquito net at night if they would be bitten in the early evening. They worried about the effectiveness and quality of condoms available and some were unsure how to use them properly. The family planning group felt that the efficacy of various contraceptive methods was reduced because people discontinued them due to worries about side effects.

#### Adoption

Some of the most thoughtful discussions in the groups focussed on why individuals did not adopt preventive measures of which they were aware. They reported a number of negative attitudes towards particular interventions. For example, they found abstinence hard and worried about their sexual instincts especially as there was little to do in their free time. Two men commented on the “sweetness” of sex without condoms. There was limited awareness of the benefits of early testing and antiretroviral treatment. Indeed, many were unclear about the distinction between HIV and AIDS. As well as the obvious implications for health, they feared the consequences of positive HIV test results, such as whether partners might stick with them and whether they would themselves be able to have children. Some were aware of reported cases where people who had been diagnosed with HIV had then spread the condition maliciously.

Negative attitudes were also important as a barrier to the adoption of family planning, with worries about side effects such as infection, disruption of menstrual bleeding and risk to future fertility being common. In particular there was a fear that any erratic bleeding must be bad for the body and that some contraceptive methods might cause miscarriage, referred to as “slippery uterus”. Although they largely accepted the need to limit family size, the traditional belief that having more children would provide labour and security in future years partly undermined messages about the benefits of smaller families. They were aware that some religious groups held that using condoms or contraception might encourage immorality and a few participants questioned whether family planning was against biblical teaching.

Conversely, they were also aware of peer pressure on young people to become sexually active, and also pressure within relationships when one partner, usually the man, wanted more children. Indeed there were reports that these disagreements sometimes led to domestic violence.

Some saw impregnated nets as uncomfortable in hot weather, and others said that the chemicals caused itching. Concerns about coils included the cost, the fumes and the risk of fire. Although families took measures to keep mosquitoes out of their houses and cut back vegetation to prevent them breeding, they were less aware of the need to deal with stagnant water or to cover compost pits. For people living in marshy areas, controlling mosquitoes seemed impossible.

Cost was also an issue, because although impregnated nets and contraceptives were available from local drugstores, the expenditure was hard for families to justify when they had limited money for basic necessities such as salt, soap or paraffin.

#### Implementation

In these discussions the young people focussed mainly on how public health messages should be conveyed, rather than how services should be organised. They identified a need for more specific information on how to prevent the transmission of HIV, as well as wider dissemination of information on the value of early testing and how effective antiretroviral treatment can be. Linked to this, they were unaware what treatment was available locally.

There was a feeling that people who lacked confidence needed help to say no, or to insist on condom use. They recognised that being faithful could be hard, but were unsure as to how people might be helped to achieve this. Because discussions about family planning were sometimes difficult between men and women, it seemed important that sensitisation programmes should engage young people of both sexes together. Although word of mouth was seen as the most important way to convey messages about each of the three topics, visual aids and the use of drama, discussion groups, advice in antenatal clinics, the church and mass media were also regarded as useful.

Later in the conference, there was an interesting exchange when one of the speakers berated the local community for not taking up the offer of free nets when these had been available in the past. This prompted murmurs of dissent and many participants asserted that the arrangements had been poorly publicised; they believed that the supply had then been sent elsewhere and sold. This highlighted the importance of effective implementation.

#### Maintenance

Less of the discussion related to maintaining healthy behaviours, although this is important if health policy is to have a lasting impact. The need to help people to avoid risk on an on-going basis was recognised to be important but not easy in HIV prevention. Mosquito nets were easily torn and needed to be replaced regularly. In the family planning group, participants tended not to distinguish between different types of contraception, and suggested that people who encountered side effects were more likely to stop using any, than to try an alternative.

### Further discussions with community leaders

In order to check we had not missed significant issues, we sought to validate the findings in discussions with community leaders after the workshop. They agreed that the issues raised were important, but also raised social problems which made people more vulnerable, making it harder for them to limit their family size and reduce their risk of HIV/AIDS. These included a concern that financial hardship might lead women into accepting gifts for sex, or prostitution. Furthermore, alcohol was seen as fuelling the risk of unsafe sex and domestic violence. Several of the community leaders also raised concerns about the quality of local health services, particularly the government-run health centres.

## Discussion

For young people in Uganda, HIV/AIDS, malaria and family planning represent important challenges that they need to address personally. Whereas tackling malaria was seen as requiring practical measures, HIV/AIDS and family planning also raised moral questions and posed a challenge to some norms of the society in which they were growing up. During the focus groups the policy of abstinence and being faithful was respected but seen as hard to follow, partly because gender imbalance and economic circumstances were seen as putting pressure on women to have sex. There were misconceptions about the effectiveness of treatment for HIV/AIDS and also the side effects of contraceptives which contributed to negative attitudes towards these interventions. Cost, rural isolation and the quality and availability of health services also limited the extent to which people were able to follow health advice.

### Strengths and weakness of this research

The way that participants were recruited and the workshop was conducted will have influenced the findings. The large number of participants, the free discussions which took place and the fact that the community leaders identified few other issues suggests that we were reasonably successful in capturing the perspectives of the young people who took part. However it was noteworthy that the male students contributed rather more than the women, suggesting some social pressure in the groups. Perhaps somewhat different findings might have emerged had it been possible to conduct individual interviews, but having these discussions in mixed sex groups may have provided a better insight into the way these issues are discussed in society more widely. Cultural factors may also have had an impact, as the groups were facilitated by Western doctors, with a female Ugandan facilitator also leading the family planning group.

Participants had been recruited through their schools and college, but this meant that children who had dropped out of school were not represented, despite these being most at risk of early pregnancy. In Kanungu, over 90% of the population are Christian, divided about equally between Anglicans and Catholics, but there are relatively few Muslims, a group who are more numerous elsewhere in Uganda. Although one Catholic school was represented, the college and most of the schools were aligned with the Anglican Church. These demographic factors may limit the extent to which specific observations might apply in other communities but the core findings that cost, misconceptions, social pressures and the conflict between human need for sexual activity and strictures to limit this are at the root of the gap between people’s good intentions and their actual behaviour seem valid more widely.

There is a growing body of literature on factors which facilitate behaviour change and the implementation of new initiatives in health [20,23,24]. Using the RE-AIM approach appeared to work well in this study; participants were able to differentiate between the different domains and this made it easier to structure the discussions about the different individual and organisational factors that influenced the uptake of the preventive measures being considered [20].

### Influences on reproductive health

National surveys confirm an imbalance between what men and women want in terms of family size, with married women in the 2006 DHS reportedly wanting 5.3 children, and men 6.4. In that survey, only 24% of married women were using any contraceptive method and many couples (45%) had not discussed contraception in the previous year. Indeed, about one in five of married women who used contraception did so without her husband’s knowledge [8]. Women in a focus group study also discussed their need for secrecy when they are under pressure to either conform to the goal of abstinence if unmarried, or alternatively conceive a large family if married [25]. It is not surprising that misconceptions and fears about the safety of contraception should persist in a society where these matters are seldom discussed. This supports the argument made by our participants for education programmes on reproductive health to engage both sexes.

In a recent focus group study, conducted in a slum area of Kampala and a nearby rural community, adolescents discussed how their sexual experience was affected by harmful cultural practices, by power imbalances (for example between younger women and older men) and by lack of information about contraception [13]. Nevertheless as in our study, the young people saw themselves as ultimately responsible for their own lives. Extending this, they saw themselves as a resource and were prepared to help others learn about reproductive health.

Amongst our participants, religious strictures promoting abstinence were seen as important but hard to follow. Similar findings emerged from a survey of University students in Mbarara which found that 37% of male and 49% of female students had not had sex, and that this was strongly associated with the importance of religion to the individual [26]. Within protestant groups, the social pressure to remain celibate appeared to limit opportunities to discuss contraception and sexual health openly, prompting the question whether emphasising abstinence protects young people, or whether it leaves them unprepared for sexual experiences that many will have anyway.

### Learning about interventions

We identified doubts about effectiveness and fears of side effects as major reasons why people did not use the different interventions discussed; condoms, antiretroviral treatment, contraceptives and impregnated nets all prompted scepticism. Our finding that misconceptions and fears are a barrier to contraceptive use echoes similar reports by Nalwadda et al. who described worries about infertility from pills which “burn the woman’s eggs”, and about condoms that are faulty, infectious or the wrong size [25].

A national survey of Ugandan adolescents found that having better knowledge of condom use was associated with the likelihood of them being used, whereas self-assessment of the risk of HIV was not, suggesting that efforts to educate young people about condoms and normalise their use are more likely to be effective than just emphasising the risk of HIV [27]. This reinforces our finding of the importance of addressing the doubts that some young people have about condoms. In contrast to our finding that knowledge about antiretroviral therapy was limited, a population study in another area (Kabarole) found this knowledge was reasonably good, suggesting that local services and information campaigns can play an important role in sensitising people [28].

Although malaria is the leading cause of mortality and morbidity in Uganda, particularly amongst pregnant women and children, it is a condition that Ugandan families have had to live with for generations. In our focus group, participants and their families relied on traditional strategies to reduce the number of mosquitoes around the home and few used nets. It is however instructive to compare these views with epidemiological reports from the nearby district of Mbarara. In 2004, 23% of children aged five or under in rural communities were using bednets, around half of which were impregnated and 43% tested positive for malaria [29]. Six years later in 2010, bednet usage had increased to 45% while the malaria prevalence had fallen to 10%. The changes in net usage may not fully explain the falling malaria prevalence, because treatment strategies have also changed, but these figures suggest that other communities are embracing malaria prevention more effectively than Kanungu. The exchange between participants and local officials later in the conference reinforced this view that local implementation of malaria prevention had been deficient.

### The role of health services

A systematic review of the utilisation of Ugandan health services suggests that an inverse care law operates in that poorer people experience a greater burden of disease but have worse access to services [6]. Distance to health facilities, perceived quality of care and the availability of drugs influence how services are utilised. Other barriers include a perceived lack of skilled staff in public facilities, the attitudes of health workers, costs of care and lack of knowledge. Misappropriation of drugs remains a major drain on the resources of the government health system, as illustrated by a recent case in which 625,000 doses of antimalarial drugs disappeared [30].

As our participants highlighted, more effective health services could provide better health education, sensitising people to ways that they can protect their own and their family’s health [31]. Although funding and research initiatives often focus on specific problems, tackling these may be more effectively achieved by generic primary care services that win people’s trust.

## Conclusions

Our findings suggest that more needs to be done to improve the implementation of health policy in rural Uganda. Young people face a series of challenges to their health; they can play an important role in addressing these, both personally and as a support to others but they need more help in doing so.

Given the immediate and serious risks that HIV, malaria and uncontrolled population growth pose, individuals need to overcome their reticence to adopt well-proven interventions and the government, foreign donors, religious organisations and other groups in civil society need to find ways to support this urgently. Specifically, the widespread lack of trust in the effectiveness of interventions such as mosquito nets, condoms, antiretrovirals and contraceptive methods needs to be addressed.

In the government health system, better measures to increase accountability and track clinical supplies to prevent their fraudulent diversion to private clinics will be important. In the non-government sector there appears to be a need to ensure the quality and consistency of information provided about health. This is particularly important for those services that are linked to faith groups that are ambivalent about going beyond the message of abstinence. Furthermore, greater thought needs to be given to maintaining initiatives – nets get torn, supplies run out and, unless updated, community leaders give out-of-date advice.

Conducting this workshop in a relatively isolated rural area highlighted the need to ensure that health education initiatives and primary care services should include outreach to smaller villages in rural areas. These should provide evidence-based information about family planning, HIV and malaria prevention which could be delivered as a multifaceted programme alongside other services such as immunisations and advice on nutrition. Ultimately, closing the gap between intentions and actions needs the various influential forces in Ugandan society to work together to help young people to protect their health in the future.

## Abbreviations

DHS: Demographic and Health Survey; RE-AIM: Reach Efficacy, Adoption, Implementation and Maintenance.

## Competing interests

The authors declare that they have no competing interests.

## Authors’ contributions

JG, CG and KS conceived this work. HT organised the conference and recruited participants. JG facilitated the malaria group; KS facilitated HIV/AIDS and CG & GK facilitated family planning. All authors met to interpret the findings. JG drafted the manuscript in discussion with the other authors who all contributed to the final manuscript. All authors read and approved the final manuscript.

## Authors’ information

JG is a General Practitioner and Senior Clinical Research Associate, Department of Public Health & Primary Care, University of Cambridge, Cambridge, UK. CG is a General Practitioner at Lensfield Medical Practice, Cambridge, UK. KS is a General Practitioner at Killick Street Health Centre, London, UK. GK is Teacher Trainer, Volunteer Uganda, Kanungu, Uganda. HT was Business Manager, Great Lakes Regional College, Kanungu, Uganda. HT sadly died of a malaria-related illness before this work was published. He was a committed and thoughtful man who could have contributed so much more to his community. The other authors are grateful to his family for their support in going ahead with publishing this manuscript.

## Pre-publication history

The pre-publication history for this paper can be accessed here:

http://www.biomedcentral.com/1471-2458/12/1022/prepub
